# Eyes in the Wild and Minds in the Loop: A Systematic Review of Artificial Intelligence for Camera-Trap Wildlife Monitoring

**DOI:** 10.3390/s26216794

**Published:** 2026-10-27

**Authors:** Areej Alsini

**Affiliations:** Department of Computer Science and Artificial Intelligence, College of Computing, Umm Al-Qura University, Makkah 24353, Saudi Arabia; aosini@uqu.edu.sa

**Keywords:** camera traps, wildlife monitoring, deep learning, computer vision, object detection, species classification, domain generalisation, edge deployment, systematic review, PRISMA 2020

## Abstract

Camera traps generate imagery faster than people can review it, and artificial intelligence (AI) is increasingly used to analyse it. This systematic review asks to what extent AI applied to camera-trap imagery has moved from benchmark performance to ecologically usable monitoring. Following PRISMA 2020, Scopus was searched on 6 August 2026 for 2020–2026. Peer-reviewed English-language journal articles applying automated inference to imagery from unattended cameras observing free-ranging animals were included; captive or web-sourced imagery, non-image methods, reviews and conference papers were excluded. Of 637 records, 231 were read in full and 201 were included. Studies were appraised against six quality criteria, findings were synthesised as descriptive counts across five research questions, and performance was tabulated on each study’s own metric, without meta-analysis. Output grew by about 29% per year. Detection and species classification each appear in 62% of studies, behaviour recognition in 5%, and study regions invert global biodiversity gradients. Only 62% of studies evaluate beyond their training data, a share unchanged since 2020; the measured generalisation penalty ranges from six to nearly seventy points. Only 35% report field deployment; costs appear only in deployment-oriented studies. The gap between benchmark performance and ecological practice remains the field’s central challenge.

## 1. Introduction

### 1.1. Camera Traps and Camera-Trap Wildlife Monitoring

Camera traps—motion-, heat- or time-triggered cameras deployed unattended to observe free-ranging animals—are a standard instrument of terrestrial wildlife monitoring. They operate continuously without an observer, suit species that are rare, nocturnal, cryptic or wary, and produce a verifiable, archivable record; camera trapping was adopted in large part to correct the detection bias of direct observation [1,2]. Throughout this review, *camera trap* denotes the instrument, and *camera-trap wildlife monitoring* denotes the practice of deploying such instruments, alone or in networks, to estimate occupancy, abundance, density, activity or community composition.

The practice has matured over three decades. Photographic capture–recapture made camera traps a density-estimation tool for individually marked species such as tigers [3]; the random encounter model extended density estimation to species without individual markings [4]; and the methods were consolidated into a common analytical framework [1,5]. Comparative studies have shown that camera traps are an effective sampling tool relative to alternative survey methods [6], and coordinated networks now operate at regional to global scales [7]. Two field studies illustrate the range of effort involved: 291 trap-nights for four photographs of a Data Deficient armadillo [8] against 3741 camera-trap nights in a single steppe inventory [9].

### 1.2. The Image-Processing Bottleneck and the Rise of Automated Analysis

The same properties make camera traps prolific. Imagery accumulates at a rate set by wildlife, weather and vegetation rather than by the research team, and false triggers inflate it further. The conventional response—every image viewed, identified and coded by a person—does not scale, is slow relative to management decision cycles, and is hard to keep consistent across observers; even large citizen-science efforts such as Snapshot Serengeti [10] distribute this burden rather than remove it. The corpus of this review carries the same signature: of the 637 records its search identified, 82 were excluded precisely because they used camera traps with no automated inference step—ecological studies whose every image was reviewed by hand (Section 2.4).

Automated analysis of camera-trap imagery predates the period reviewed here. Early systems combined hand-crafted image features with classical classifiers [11]; convolutional neural networks were first applied to camera-trap species recognition in the mid-2010s [12,13]; and between 2018 and 2019, deep learning reached accuracies comparable to human volunteers on large benchmarks [14,15,16], object detectors were adapted to camera-trap data [17], and shared detection pipelines such as MegaDetector [18] began to move automated filtering into routine practice. The same period produced the first warning about transfer: models trained at one set of locations performed markedly worse at new ones [19]. These pre-2020 studies established the approaches—feature engineering, deep classification, detection-then-classification and citizen-science integration—on which the literature reviewed here builds; they are therefore summarised here as background rather than synthesised.

### 1.3. Previous Reviews and the Knowledge Gap

Several reviews have mapped parts of this territory. Weinstein [20] and Christin et al. [21] surveyed computer vision and deep learning across animal ecology; Schneider et al. [22] reviewed computer vision approaches to individual re-identification from camera-trap data; Tuia et al. [23] set out the opportunities and obstacles of machine learning for wildlife conservation; and Vélez et al. [24] compared the platforms that deliver camera-trap AI to practitioners. Three points of consensus emerge from this body of work: deep learning performs well on the data distribution it is trained on, automation can reduce the human labour of image review substantially, and progress depends on collaboration between ecologists and computer scientists. One finding remains in open conflict: some studies report steep losses when models meet new locations [19], whereas classifiers trained on broad, multi-site data have been reported to transfer acceptably to new locations (Section 3.4).

What none of these reviews provides is a systematic, quantified account of whether the systems now being built hold up outside the conditions that trained them, and whether they reach ecological practice. Earlier reviews were narrative rather than PRISMA-based, predate the rapid growth in output since 2020 (Section 3.1), and precede the arrival of transformer and vision–language models. In particular, no previous review has measured how often generalisation is tested, how large the penalty is when it is tested, what recovers it, or what field deployment costs in accuracy, hardware, labour and money. This is the knowledge gap that justifies the present review.

### 1.4. Core Question, Research Questions and Contributions

This review addresses a single core question: to what extent has artificial intelligence applied to camera-trap imagery moved from benchmark performance to ecologically usable monitoring? The title expresses its two halves. The eyes in the wild are the camera traps and the imagery they produce; the minds in the loop are the models that interpret that imagery and the ecologists who verify, adopt or reject their outputs. The literature sits at the junction of two communities—ecology and computer vision—that publish in different venues and mean different things by a result: a benchmark improvement in one, a change in what an ecologist can infer from a season of imagery in the other.

The primary objective is to map and critically appraise, in accordance with PRISMA 2020 [25], the peer-reviewed literature that applies automated inference to camera-trap imagery from 2020 onwards. Five research questions decompose the core question: RQ1 establishes the context (growth and venues), RQ2 characterises what is built (architectures and tasks), RQ3 where and on what it is built (taxonomic, geographic and ecosystem coverage), RQ4 whether it holds beyond its training data (evaluation rigour), and RQ5 whether it reaches practice (field deployment and ecological use). RQ1 is answered from all 637 identified records, because growth and venue structure are properties of the whole literature; RQ2–RQ5 are answered from the 201 included journal articles. The scope is camera-trap imagery only, as defined in Section 2.3; acoustic, telemetry, uncrewed aerial, thermal-only, footprint and seismic monitoring are excluded so that modality-specific questions about evaluation and deployment remain answerable. No taxonomic or document-type restriction is applied at search, so absences under RQ3 are properties of the literature, not of the query.

Beyond updating the descriptive map of the field, this review makes four contributions not available in earlier reviews:

1. a quantified, cross-study account of the generalisation penalty, reporting within- and beyond-training performance for 28 systems on each study’s own metric, together with the measured cost of each recovery (Section 3.4);
2. a systematic ledger of field-deployment costs—accuracy lost on edge hardware, degradation in the field, energy, human hours and money—compiled from 24 deployed or deployment-oriented systems (Section 3.5);
3. evidence that the share of studies testing beyond their training data has not risen since 2020 (Section 3.4);
4. evidence that the publication base is shifting toward conference proceedings, from 30% of annual output in 2020 to 41% in 2025, with direct consequences for how future reviews must be designed (Section 3.1).

Section 2 reports the methods, Section 3 the results, Section 4 the discussion, Section 5 the limitations, and Section 6 the conclusions.

## 2. Materials and Methods

This review was conducted and reported in accordance with the PRISMA 2020 statement [25]. The PRISMA principles are not restated here; this section reports only how they were implemented in the present review. The workflow ran in seven stages: formulation of the research questions (Section 2.1); a structured database search (Section 2.2); application of pre-specified eligibility criteria (Section 2.3); title-and-abstract screening and full-text selection (Section 2.4); data extraction (Section 2.5); quality appraisal of the included studies (Section 2.6); and synthesis (Section 2.7). This review was not registered and no protocol was published; all definitions, reason categories and operational checks were fixed before full screening began.

### 2.1. Research Questions

The five research questions below decompose the core question stated in Section 1.

- RQ1. Has research on AI-assisted camera-trap monitoring grown between 2020 and 2026, and how is output distributed across venues and research communities?
- RQ2. Which model architectures and monitoring tasks characterise the field, and how has that composition shifted?
- RQ3. Which taxa, regions and ecosystems are represented, and where are the blind spots?
- RQ4. How rigorously are these systems evaluated—do studies test generalisation across sites, seasons and camera configurations, or report only within-dataset performance?
- RQ5. Do these systems reach ecological use and field deployment, and what constrains the gap between benchmark and practice?

### 2.2. Information Sources and Search Strategy

The electronic database Scopus was the information source. Scopus was chosen because it indexes both of the field’s parent literature—the ecological and conservation journals and the computer science journals and major computer vision conference proceedings—within a single interface, and because it exports, for every record, the structured metadata (document type, source title, citation count and open-access status) on which the growth and venue analysis of RQ1 depends. The consequences of searching a single database are discussed in Section 5.

The search was built from two concept blocks (Table 1). The first block captures the instrument and is a required concept, so that every retrieved record concerns camera traps; it includes spelling variants and the term *trail camera*, which is common in North American and hunting-derived literature. The second block captures automated analysis at three levels of generality: field-level terms (deep learning, machine learning, computer vision, artificial intelligence), method-family terms (neural network, convolutional, YOLO, transformer), and generic terms (automated, automatic) that retrieve pipelines not described in machine learning vocabulary. The two blocks were combined with the Boolean operator AND, terms within each block with OR, and the truncation symbol (*) captured plural and derived forms.

The search proceeded in five steps. (1) The query was entered in the Scopus Advanced Search interface, restricted to the title, abstract and author-keyword fields (TITLE-ABS-KEY). (2) The publication-year limit (2020–2026) and the English-language limit were applied; no taxon, subject-area or document-type limits were applied, and conference proceedings were retained. (3) The search was executed on 6 August 2026 and returned 637 records. (4) All 637 records were exported with full bibliographic metadata and abstracts. (5) The export was checked for duplicate records (none were found, as expected from a single-database search) and passed to screening (Section 2.4). The complete search string, as executed, was:

TITLE-ABS-KEY ( “camera trap*” OR “camera-trap*” OR “trail camera*” OR “camera trapping” ) AND TITLE-ABS-KEY ( “deep learning” OR “machine learning” OR “computer vision” OR “artificial intelligence” OR “neural network*” OR “convolutional” OR “YOLO” OR “transformer” OR “automated” OR “automatic” ) AND PUBYEAR > 2019 AND PUBYEAR < 2027 AND ( LIMIT-TO ( LANGUAGE , “English” ) )

### 2.3. Eligibility Criteria

**Figure 1:**
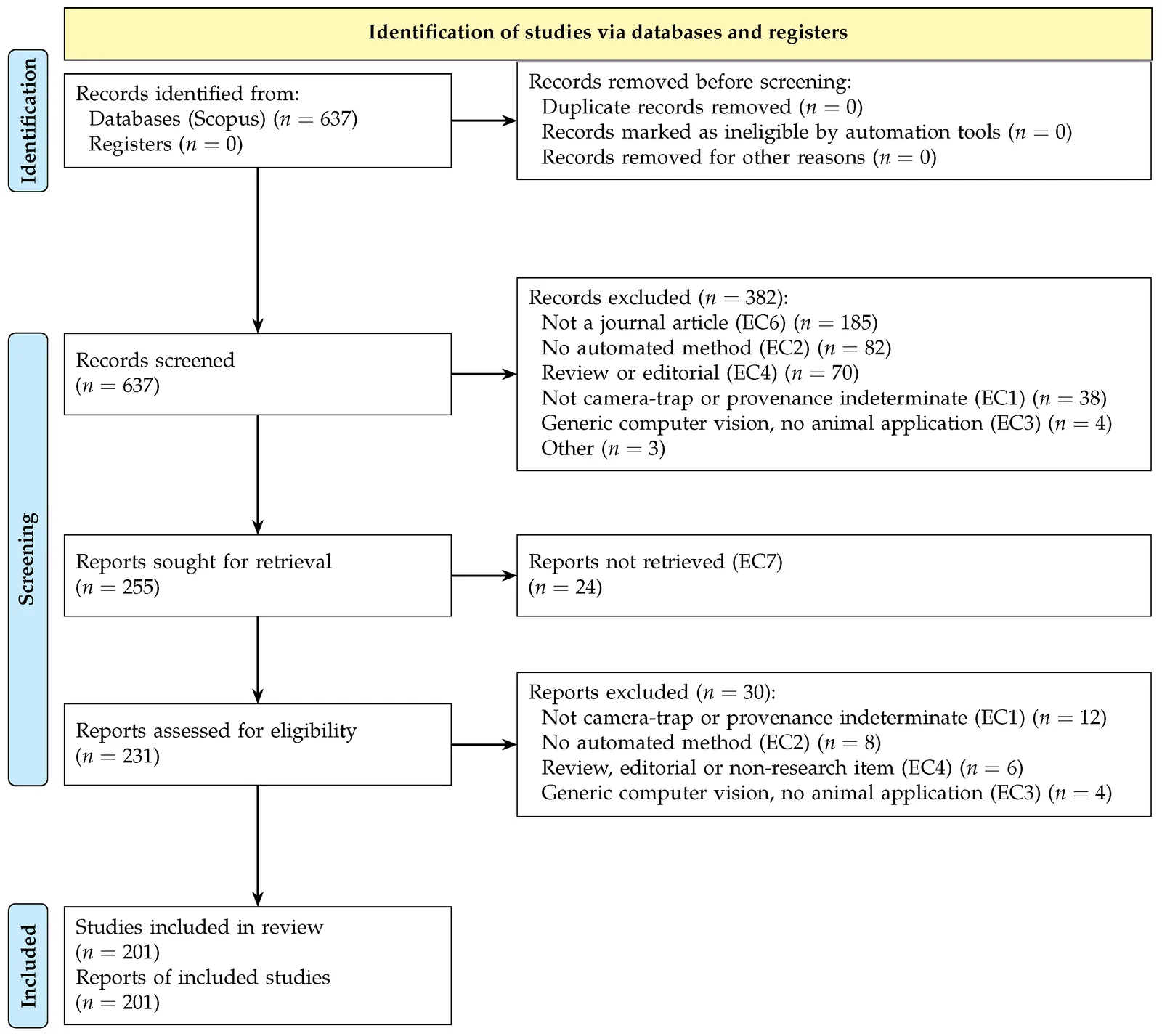
PRISMA 2020 flow diagram, following the official template for new systematic reviews that searched databases and registers only [25]. Exclusion reasons are mapped to the criteria codes of Table 2; AI pre-classification excluded no record without human verification (Section 2.4). The 201 included studies are the denominator for Section 3.2, Section 3.3, Section 3.4, Section 3.5 and Section 3.6.

Studies were eligible where they applied an automated or computational inference step to camera-trap or trail-camera imagery. The inclusion criteria (IC1–IC6) and exclusion criteria (EC1–EC7) are set out in Table 2; each exclusion recorded in Figure 1 corresponds to one of these codes. Three operational rules govern the borderline cases. A camera trap is an unattended, fixed camera—motion-, heat-, time- or image-content-triggered – observing free-ranging animals. An automated inference step is an algorithm that decides what an image contains; image management, independence filtering and triggering hardware do not qualify, and neither does machine learning operating downstream of manually identified image content, such as species-distribution models or density estimation from manually classified records. Where a record’s imagery provenance cannot be established (web-collected, museum or curated photographs presented under camera-trap vocabulary), the indeterminate-provenance rule excludes it, and full-text provenance checking is mandatory because the indexed fields cannot reveal the problem.

The start year of 2020 was chosen so that the review captures the period in which deep learning became the dominant approach and in which output grew most rapidly (Section 3.1); the foundational pre-2020 studies are summarised in Section 1 as background. Synthesis (RQ2–RQ5) is restricted to peer-reviewed journal articles, so that every synthesised study reports its methods at full length; the growth analysis of RQ1 uses all identified records. Journal indexing status and quartile were not used as eligibility criteria: every record is Scopus-indexed by construction, eligibility was decided on study content, and a quartile describes the publication venue rather than the quality of an individual study, which is appraised separately (Section 2.6).

### 2.4. Selection Process

Records were screened on title and abstract and coded Include, Maybe or Exclude, with a stated reason. Screening was performed by a single reviewer (the author), assisted by an AI pre-classification of every record. The reviewer verified every Include and Maybe code, and seeded 10% audits of both sides—16 eligibility exclusions and 25 verified Includes—found no decision disagreement, against a pre-set rule that three or more errors would trigger a full re-screen. All definitions, reason categories and operational checks, including the indeterminate-provenance rule and a requirement that every retrieved document be checked against its record on title, authors and venue, were fixed before full screening and applied uniformly.

Every provisional decision from the AI-assisted pass was verified by the author against the sharpened definitions. Forty-one records initially coded Maybe were resolved (16 Include, 25 Exclude); ten provisional Includes were reversed through four targeted checks (non-image modalities, drifting imagery provenance, downstream-only machine learning and generic computer vision); and one record initially excluded as provenance-indeterminate was reinstated once its full text was obtained. No record was excluded by the automation tool alone. The verified title-and-abstract outcome, reported in Figure 1, is 255 records sought for retrieval and 382 excluded: not a journal article (EC6), 185; no automated method (EC2), 82; review or editorial (EC4), 70; not camera-trap or provenance indeterminate (EC1), 38; generic computer vision with no animal application (EC3), 4; other, 3. Of the 255 reports sought, 24 could not be obtained (EC7); all 231 retrieved reports were read in full, and 30 were excluded at that stage, leaving 201 included studies. Ambiguity was resolved conservatively throughout.

### 2.5. Data Extraction

From every included study, the same fourteen items were recorded by the author (Table 3), chosen so that each research question has exactly the evidence it needs. Categorical items are recorded in a fixed vocabulary; metrics, locations and notes are recorded verbatim as free text; and *not reported* is recorded explicitly, because how often a study is silent on a field is itself a result. The generalisation test is coded as the strongest design reported, on an ordered scale from a same-site random split through temporal holdout, cross-camera and cross-site evaluation to cross-dataset transfer.

### 2.6. Quality Appraisal

**Figure 2:**
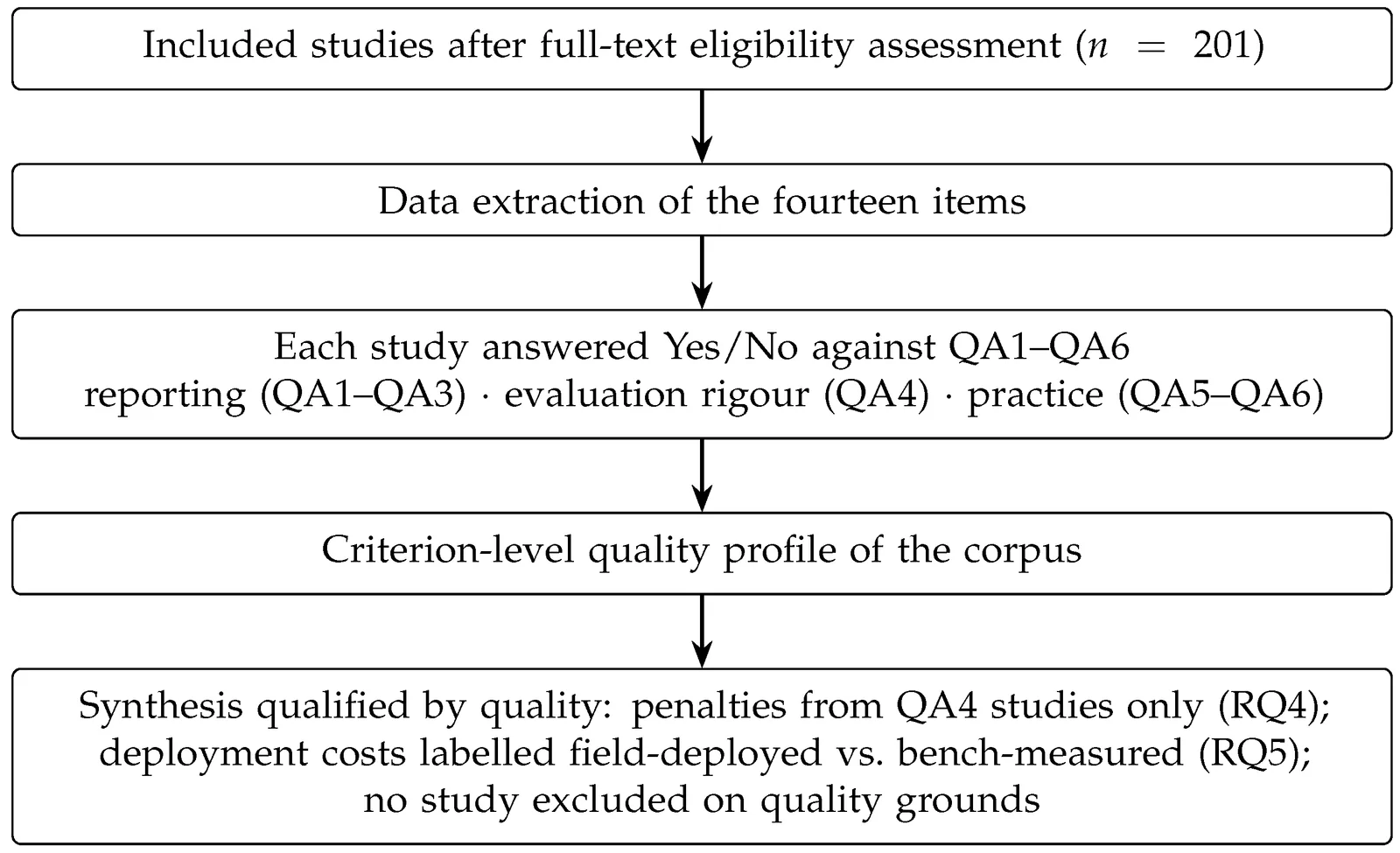
Quality-appraisal procedure applied to the included studies.

Every included study was appraised against six quality criteria (QA1–QA6, Section 3.6, each answered Yes or No from the extraction items of Table 3. The criteria were framed around the research questions: two concern whether the study is reported fully enough to be reproduced and situated (QA1, QA2), one whether its computing requirements are stated (QA3), one whether its performance claims are tested beyond the training data (QA4), and two whether it connects to ecological use and field practice (QA5, QA6). Figure 2 summarises the procedure.

Quality appraisal was used to qualify the synthesis, not to exclude studies. Excluding studies that fail QA4 or QA6 would remove precisely the evidence that RQ4 and RQ5 are designed to measure and would make the field appear more rigorous and more deployed than it is. Instead, the appraisal determines which studies contribute to which conclusions: generalisation penalties (Section 3.4) are drawn only from studies meeting QA4, and the deployment costs of Section 3.5 are reported with an explicit statement of whether each system was field-deployed or bench-measured. Formal risk-of-bias tools designed for intervention or diagnostic-accuracy studies were not applied, because the included studies do not estimate treatment effects and the review does not pool effect estimates; the criterion-level results are reported in Section 3.6.

### 2.7. Synthesis Methods

Because the included studies address different tasks, on different datasets, with different performance metrics, a meta-analysis was neither feasible nor meaningful. The synthesis is therefore descriptive and narrative, organised by research question. Each research question is answered with counts and percentages over the relevant denominator (637 records for RQ1, 201 studies for RQ2–RQ5); task, architecture, taxon and region categories may overlap within a study, whereas evaluation design, deployment status, compute target, data source and ecological use are coded as single exclusive categories. Growth is summarised as the compound annual growth rate over the six complete years (2020–2025); 2026 is reported as a partial year. Where studies report performance within and beyond their training data, both values are tabulated on the study’s own metric rather than converted to a common scale, and deployment evidence is tabulated in the units each study reports. The narrative synthesis then identifies the patterns, exceptions and explanations that recur across these tabulations.

## 3. Results

### 3.1. RQ1: Research Growth and Publication Landscape

**Figure 3:**
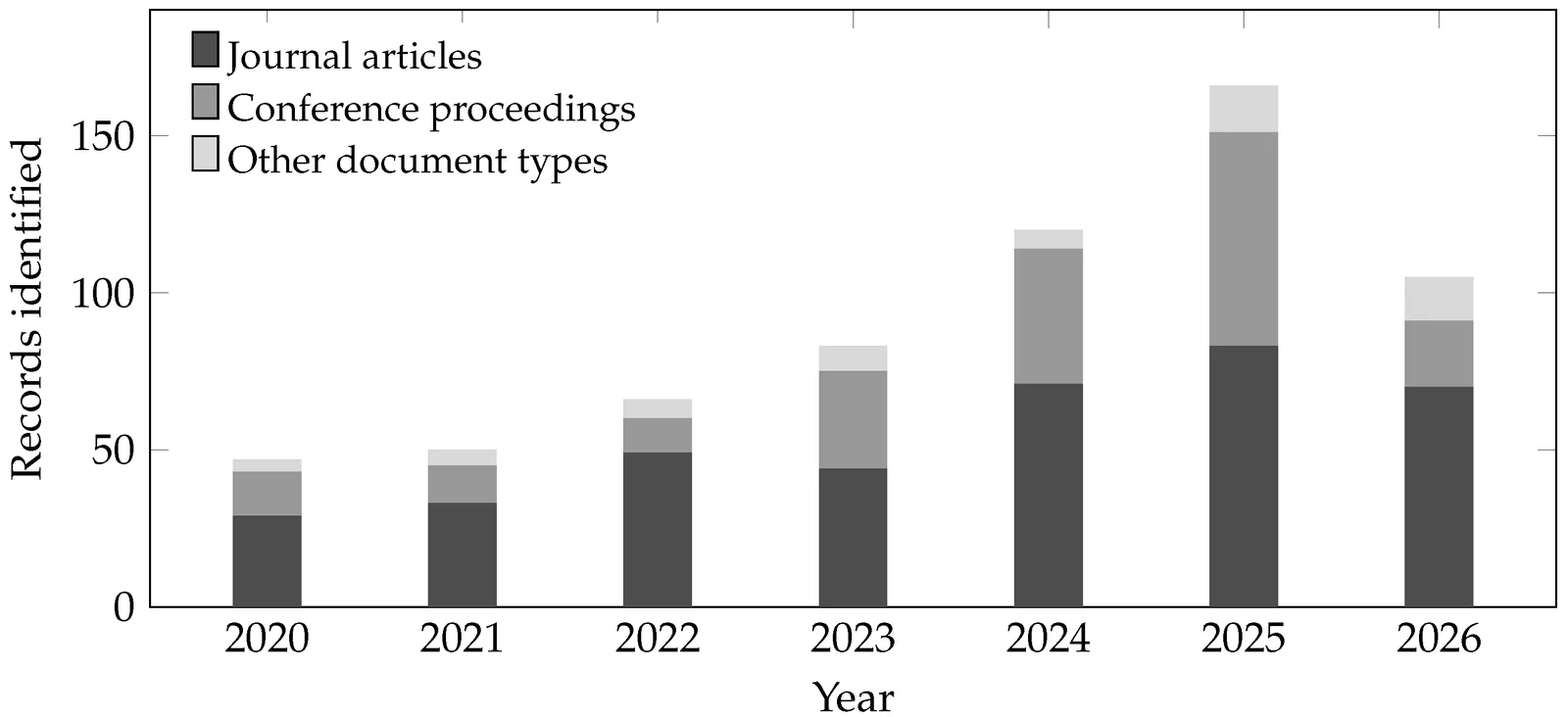
Annual publication output on AI-assisted camera-trap monitoring, 2020–2026. The 2026 column is a partial year, covering approximately seven months.

RQ1 concerns the trajectory of the field and is answered from all 637 identified records, before eligibility screening; all quantities were recomputed from the corpus export. Output rose from 47 records in 2020 to 166 in 2025 (Table 4; Figure 3), a 3.5-fold increase equivalent to a compound annual growth rate of about 29%, monotonic across every complete year; the partial 2026 count of 105 records over roughly seven months annualises to 175–180, consistent with continued growth. Conference proceedings account for 200 records and their share of annual output rose from 30% in 2020 to 41% in 2025—a substantive finding: the field’s centre of gravity is shifting toward the computer vision publication culture, so reviews restricted to journals will under-represent methodological work, increasingly over time. The corpus is dispersed across 340 venues with no dominant outlet: *Ecological Informatics* leads with 49 records (7.7%), ahead of *Methods in Ecology and Evolution* (26), *Ecology and Evolution* (21), *Remote Sensing in Ecology and Conservation* (18) and *Animals* (17), alongside computing venues. This is the structural signature of a field with two parent communities. Of the 637 records, 356 (56%) are open access; citations total 7973 (median 3, maximum 940), and 187 records are uncited, which is unsurprising given that 271 were published in 2025–2026.

### 3.2. RQ2: Architectures and Monitoring Tasks

Every count in Section 3.2, Section 3.3, Section 3.4, Section 3.5 and Section 3.6 refers to the 201 studies included in the review [26,27,28,29,30,31,32,33,34,35,36,37,38,39,40,41,42,43,44,45,46,47,48,49,50,51,52,53,54,55,56,57,58,59,60,61,62,63,64,65,66,67,68,69,70,71,72,73,74,75,76,77,78,79,80,81,82,83,84,85,86,87,88,89,90,91,92,93,94,95,96,97,98,99,100,101,102,103,104,105,106,107,108,109,110,111,112,113,114,115,116,117,118,119,120,121,122,123,124,125,126,127,128,129,130,131,132,133,134,135,136,137,138,139,140,141,142,143,144,145,146,147,148,149,150,151,152,153,154,155,156,157,158,159,160,161,162,163,164,165,166,167,168,169,170,171,172,173,174,175,176,177,178,179,180,181,182,183,184,185,186,187,188,189,190,191,192,193,194,195,196,197,198,199,200,201,202,203,204,205,206,207,208,209,210,211,212,213,214,215,216,217,218,219,220,221,222,223,224,225,226], the final box of Figure 1. All counts derive from the full-text extraction fields of Section 2.5. Task, architecture, taxon and region categories overlap, because a study may address several tasks or name several regions, so those counts do not sum to 201; evaluation design, deployment status, compute target, data source and ecological use are coded as single exclusive categories per study. RQ2 is answered along three separate dimensions: the monitoring tasks addressed (Section 3.2.1), the model architectures used (Section 3.2.2), and the tools and datasets contributed (Section 3.2.3).

#### 3.2.1. Task Distribution Characteristics

**Figure 4:**
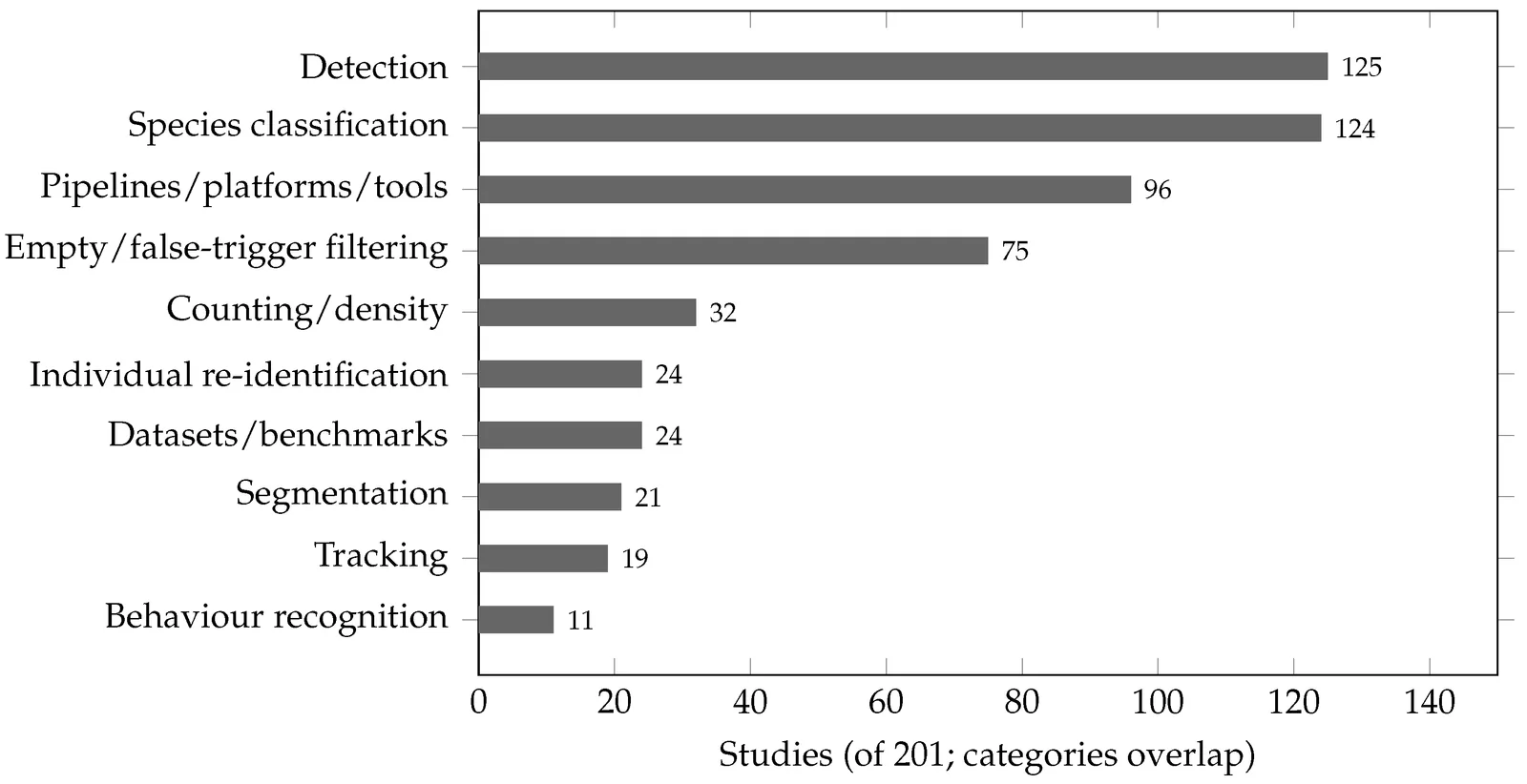
Monitoring tasks and contribution types documented at full text in the 201 included studies (RQ2). Categories overlap, so counts do not sum to 201. Behaviour recognition, the task closest to ecological mechanism, is the rarest task, an order of magnitude below detection and species classification.

Each study was coded to the monitoring tasks shown in Figure 4. *Detection*: locating animals in the frame, as bounding boxes or image-level presence. *Species classification*: assigning a taxon label to an image or detection. *Counting/density*: enumerating individuals or estimating abundance from imagery. *Tracking*: linking the same animal across video frames or image sequences. *Individual re-identification*: distinguishing individuals of one species. *Segmentation*: pixel-level delineation of the animal. *Empty/false-trigger filtering*: discarding blank or falsely triggered images before analysis, coded as a task in its own right, beyond the eligibility vocabulary of Section 2.3, because a substantial body of work addresses it directly. *Behaviour recognition*: labelling what the animal is doing.

Detection and species classification dominate: 125 of the 201 studies (62%) implement detection and 124 (62%) species classification (e.g., [42,93,135,178]). Empty or false-trigger filtering is the third most common task, at 75 studies (37%), usually as a preprocessing stage of a detection or classification pipeline (examples spanning filtering and segmentation: [64,132,177,201]). The secondary tasks follow: counting or density estimation appears in 32 studies (16%; e.g., [61,118,148,176,202]), individual re-identification in 24 (12%; e.g., [129,145,167,179]), segmentation in 21 (10%) and tracking in 19 (9%; e.g., [95,125]). Behaviour recognition is the rarest task, at 11 studies (5%; e.g., [38,134,207,221]), an order of magnitude below the two dominant tasks. The task profile is therefore steeply graded: the tasks that answer *what is present* are mature, whereas the tasks that answer *how many individuals* and *what they are doing*—the tasks closest to population and behavioural ecology—remain a small minority.

#### 3.2.2. Architecture Families and Reported Performance

The architecture is almost always identifiable: 192 of 201 studies (96%) name at least one model, and only 9 (4%) describe automated inference without identifying it (Table 5). Generic convolutional backbones (130 studies) lead, followed by the R-CNN family (82), the YOLO family (64; [69,110]), transformers (34), classical machine learning or computer vision methods (32) and vision–language or foundation models (7; [115]). MegaDetector is named in 52 studies, about one in four; because it is itself a Faster R-CNN, it accounts for most of the R-CNN family’s count. The families form a recognisable development pathway, from classical pipelines and generic convolutional classifiers, through two-stage (R-CNN) and one-stage (YOLO) detectors that localise animals before classifying them, to transformer backbones and, most recently, vision–language models that can be queried without task-specific training.

Full-text reading sharpens three of these families. The vision–language records agree that general-purpose models do not transfer to camera-trap recognition unaided: zero-shot CLIP retrieves Snapshot Serengeti attributes at 0.29 mean average precision, lifted to 0.60 by domain fine-tuning but still below a vision-only classifier’s 0.68 [115], and an instruction-tuned vision–language model applied directly achieves near-perfect precision at recall as low as 0.07–0.20, so the working system delegates recognition to a YOLOv10 detector [114]. The verdict reverses for behaviour: a multimodal large language model applied zero-shot to three-way behaviour classification reaches 86.39% macro F1, at the price of prompt fragility (bare labels collapse SigLIP from 85.67% to 10.57%) [164]. Metadata fusion pays only where context is class-informative: temperature–date–location vectors lift a ResNet50 from 98.4% to 98.9%, while the same study’s attention-fusion variant falls below its image-only baseline [142]; temporal fusion concentrates its gain in species with learnable activity rhythms [123]; and camera-identity co-supervision lifts cross-location macro F1 by 3.1 points on iWildCam, the gain tracking measured species–location mutual information and concentrating in rare classes [187].

Table 6 compares the performance that representative modern methods report in the included studies. Because each study uses its own data and metric, the table supports comparison of patterns rather than a ranking of models. Three patterns are consistent. First, within the data distribution on which they were trained, all modern families report high performance, between about 80% and 99% on accuracy-type metrics. Second, the difference between the within- and beyond-training performance of the same system is generally larger than the difference between families; in the one study that compared six architectures under the same cross-dataset test, the hybrid detector–transformer lost the fewest points [210]. Third, compact convolutional and YOLO models retain most of their accuracy when quantised for edge hardware [126,198], whereas vision–language models are not yet competitive for zero-shot species recognition but are already competitive for behaviour [115,164].

#### 3.2.3. Tool and Dataset Development

Two further categories code the contribution rather than the task: *datasets/benchmarks*, where the contribution is imagery data or an evaluation benchmark, and *pipelines/platforms/tools*, where it is a software system that wraps detection or classification capabilities for practical use. Almost half the corpus (96 studies, 48%) delivers a pipeline, platform or tool, whereas 24 (12%) contribute a dataset or benchmark (Figure 4). MegaDetector, named in 52 studies, is the field’s de facto shared detection pipeline [116], and much tool development consists of placing a species classifier, a user interface or an ecological analysis downstream of it. A further 15 studies address the annotation bottleneck directly, through self-supervised, semi-supervised, few-shot, active or continual learning [43,83,189]. The volume of tool development contrasts with its uptake: of the 111 studies whose contribution is a dataset, benchmark, pipeline or tool, 55 never leave archived imagery (Section 3.5).

### 3.3. RQ3: Taxonomic, Geographic and Ecosystem Coverage

**Figure 5:**
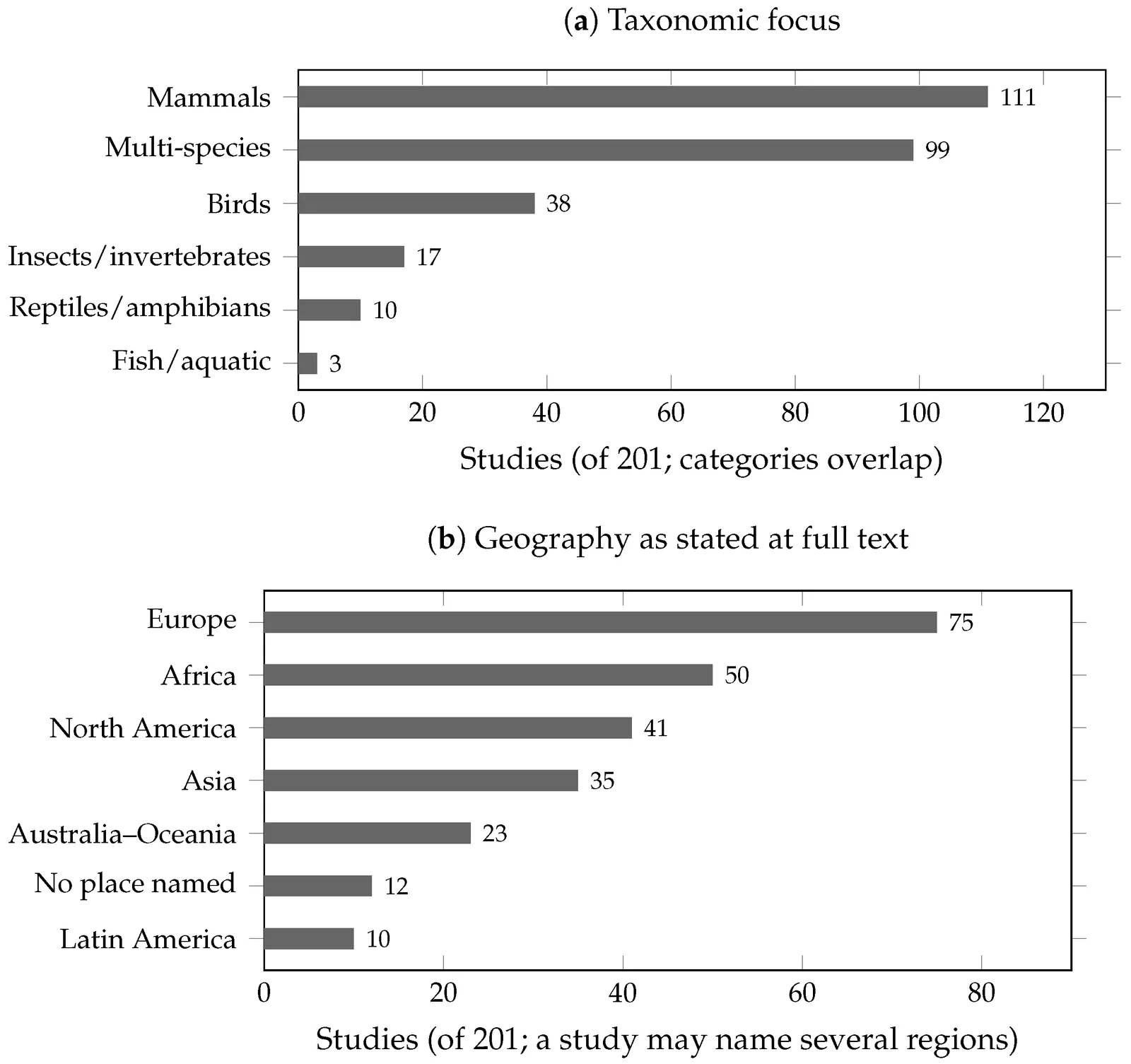
Coverage of the 201 included studies (RQ3). (**a**) Taxonomic focus. (**b**) Region of image collection as stated at full text; the distribution inverts global biodiversity gradients.

The taxonomic centre of gravity is mammalian: 111 studies (55%) work on mammal taxa and 99 (49%) on multi-species or community-level monitoring (the two overlap heavily, since most multi-species studies are mammal-dominated), against 38 (19%) on birds [141], 17 (8%) on insects or invertebrates [76], 10 (5%) on reptiles or amphibians [75,165] and 3 on fish or other aquatic taxa (Figure 5a). Full-text reading locates part of the cause in the trigger hardware: passive-infrared sensors fire on surface temperature contrast, so ectotherms can pass unrecorded, and the study that closed the gap did so by replacing the instrument—27 sites of always-on video cameras, whose million-plus frame-differenced videos, screened with YOLOv5, produced an 11,458-video dataset dominated by frogs (28.7%), spiders, lizards and scorpions [165].

Geography is stated in almost every study: 189 studies (94%) state where their imagery was collected, and only 12 (6%) present themselves as geographically unanchored methods work evaluated on unlocated or synthetic material. Where a place is stated, the distribution inverts biodiversity gradients (Figure 5b): Europe alone (75 studies) outnumbers Asia (35), Australia–Oceania (23) and Latin America (10) combined; North America adds 41 more; and the Neotropics, the most species-rich region on Earth, appear in one study in twenty. Africa (50) owes nearly half of its count (23 studies) to the Snapshot Serengeti benchmark and its descendants rather than to a broad continental literature. Independent project-level inventories report the same pattern [227].

### 3.4. RQ4: Evaluation Rigour and Generalisation

**Figure 6:**
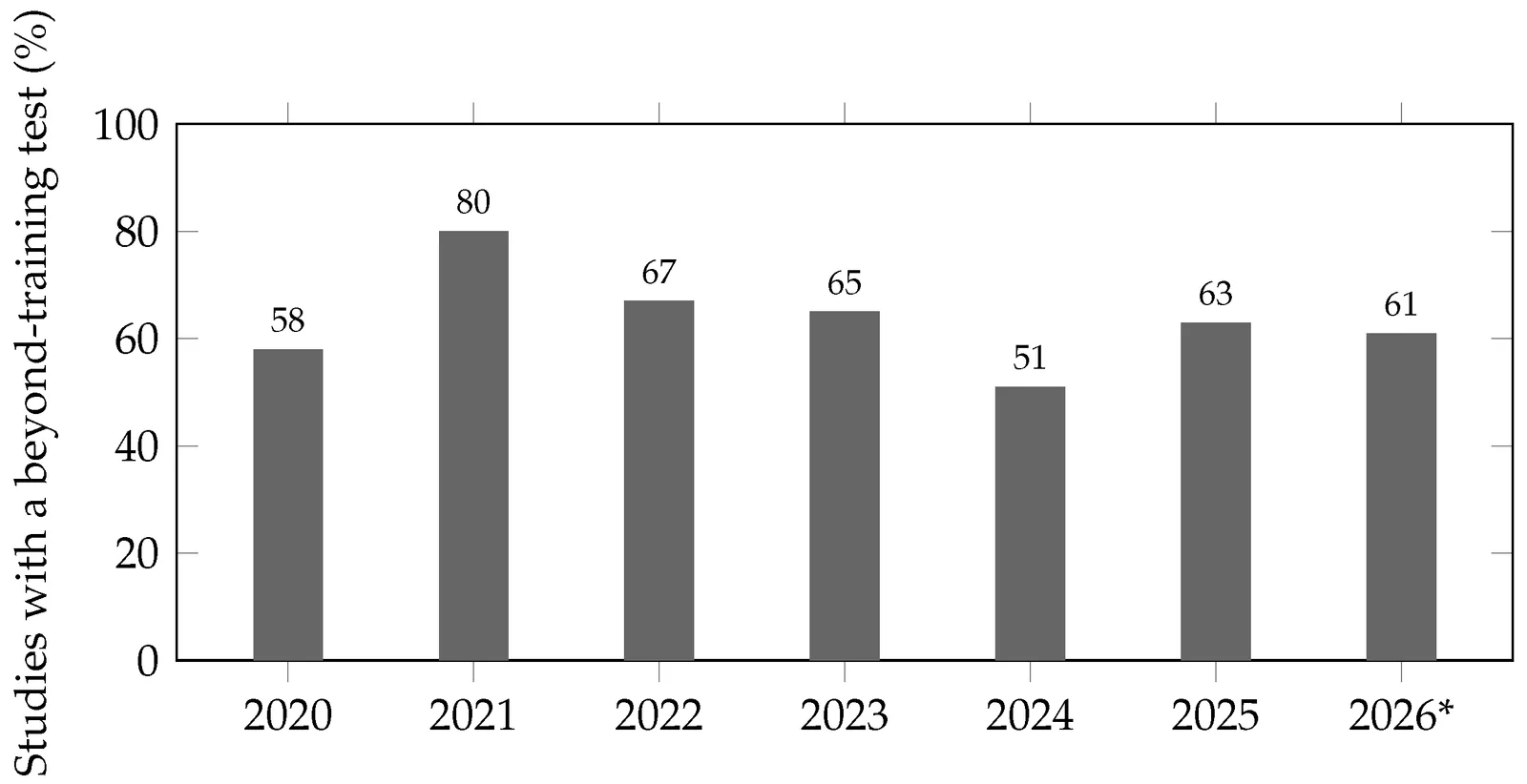
Share of included studies per year that report any evaluation beyond the training data (cross-site, cross-dataset, temporal or cross-camera holdout).

For an ecologist, the rigour question is concrete: a model that scores well on the images it was trained around may still fail at the next valley, the next season, or the next camera model, and only a test on new material can tell. The corpus is far from uniform on this point. 124 of 201 studies (62%) report at least one evaluation beyond the training data: 61 hold out whole sites or locations [34,49,96,135], 39 test on a different dataset [36,215], 15 hold out a later period [42] and 9 hold out cameras within a site [92,94]. The remaining 61 (30%) evaluate only on a random split of the same material, and 16 (8%) report no quantitative evaluation of generalisation at all. The share has not moved: it lies between 51% and 80% in every year from 2020 to 2026 with no monotonic trend (Figure 6), and it is the same among field-deployed systems (42 of 70) as among archived-imagery studies (69 of 114). What follows is what the studies that did test it found; Table 7 holds the full evidence and per-study numbers.

#### 3.4.1. The Drop, Its Exceptions and Its Cause

**Figure 7:**
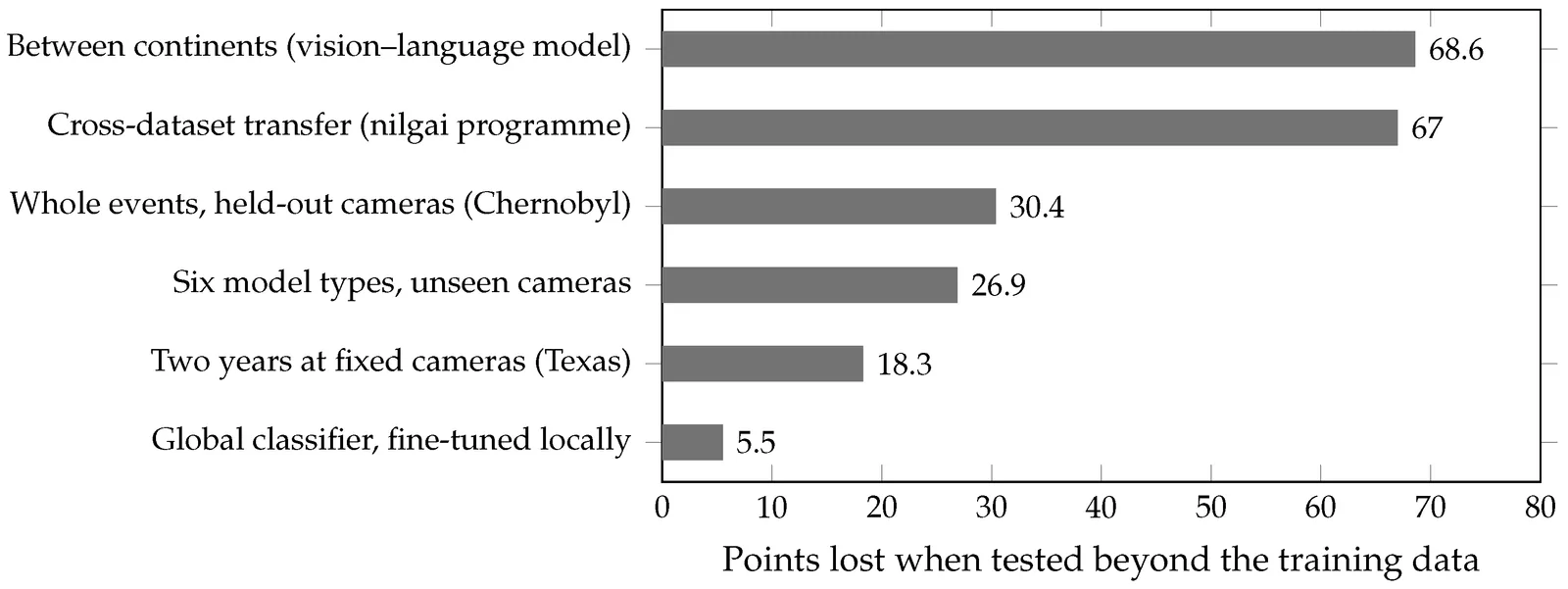
The generalisation penalty at a glance: how many points each of six systems loses when tested beyond its training data, on that system’s own 0–100 metric [34,40,42,92,212,215]. The loss ranges from about six points (a global classifier fine-tuned on local data) to nearly seventy (cross-continental transfer of a vision–language model); the within- and beyond-training values for every case are given in Table 7. The values demonstrate the magnitude of the performance drop only: because the systems are evaluated with different metrics (accuracy, F1, mAP, MCC), absolute performance cannot be compared across systems.

Where studies test on new material, the drop is consistent and often severe. Figure 7 shows, for six comparable cases, how many points each system loses beyond its training data: unseen cameras cost a six-model comparison about a quarter of its accuracy [34], a cross-continental transfer almost everything [215], with other spatial cases in between [40,94]; time alone erodes fixed-site classifiers as the vegetation behind the animals changes [42,169]; and per-image scores flatter: one detector that was respectable per image got the whole capture event right only half the time at held-out cameras [92]. The exceptions share one property, training breadth: classifiers built on imagery from many places have passed demanding field checks at hundreds of unseen stations [49,96,135], and off-the-shelf MegaDetector matches manual detection indices in regions it was never tuned for [57,127], while at the same new site, a single-location-trained detector performs no better than chance [181]. The remaining limits lie in how images are made, not where: time-lapse animals too small for the model [59,62], failed triggers [182], taxa absent from pretraining [175]. Much of the drop has one intuitive cause: the camera never moves, so the model learns the scenery behind the animal, and recognising the trees works until it meets a new forest. One identifier jumped from 57% to 95% the moment the animal was cut out of the frame [170], others were caught matching backgrounds directly [77,99], and training with animals pasted onto varied backgrounds measurably shifts attention onto the animal [104,138,174,184].

#### 3.4.2. Fixes, Honest Measurement and the Ecology Downstream

The recoveries are cheap in labels and reported with their price tags: adapting a broad existing model with a few hundred local images per species beats both the untouched global model and a locally built one [212]; scheduled or drift-triggered retraining restores decayed classifiers and cuts manual review severalfold [42,80]; and very small doses of targeted effort—five hundred local boxes, fifty annotations, an untested confidence threshold finally tuned, an on-camera filter that re-calibrates itself in minutes—buy large recoveries [79,90,143,177]. Each fix carries a stated cost, from multiplied false alarms to an overfitting boundary [180], and specialised levers exist for the data floor, hard-to-see animals and newly arriving species [70,72,103]. Reported accuracy, in turn, means little until one knows what was held out: honest designs keep whole cameras or locations out of training [85,108,121], whereas leaked splits let a detector score 96% on paper and 26% in the field [208]; some cross-dataset claims test the recipe rather than the model, or internet photographs rather than deployments [52,59,83,146,162]; training at the survey’s own empty-image ratio beats the field’s default rebalancing [51]; confidence scores need calibrating [163]; and headline accuracy hides the rare classes ecologists most care about [85,108]. Whether any of this matters ecologically depends on the estimator, not on automation: occupancy and density models absorb label error rates up to about 20%, matching expert-label results across millions of images [36,96,205,218]—until errors arrive faster than the pooling window can soak up, at which point the same machinery biases occupancy by up to +166% while a semi-automated design preserves both the inference and 97.7% of the labour saving [124,148,183,199,202]. The spread in Table 7—from a six-point drop to a near seventy-point collapse, with recoveries priced in each currency—is why a single headline accuracy is not an adequate summary of any system in this corpus.

### 3.5. RQ5: Field Deployment and the Benchmark–Practice Gap

The last question is whether all of this leaves the laboratory. For the most part, it does not: 114 of 201 studies (57%) evaluate on archived imagery only, 17 (8%) release a software tool without a field trial, and 70 (35%) report a prototype or field trial (33) or an operational deployment (37). Computing on or near the camera is described in 41 studies (20%; [67,73]), cloud inference in 19, a workstation or server in 112, and 29 do not state their compute target at all. Of the 111 studies whose contribution is a dataset, benchmark, pipeline or tool (the union of the two RQ2 contribution categories, overlapping in nine), 55 never leave archived imagery—the benchmark–practice gap in one number. Connections to ecological end-use are stronger than deployment: 117 studies (58%) link their outputs to an ecological analysis (91; occupancy, density, abundance or activity) or to a management or conservation decision (26; [90,159,214,218]), while 84 (42%) stop at the classification metric. Most studies work on their own imagery (116, 58%) or combine it with a public benchmark (47); only 37 (18%) rely on public benchmarks alone. Human-in-the-loop and citizen-science workflows [84,133,165,204] are the shape in which automation most often reaches practice. What the deployed minority reports, assembled in Table 8, differs in kind from the benchmark literature: degradation, resource cost and human hours appear as first-class results—while even a study whose stated purpose is edge deployment can end up evaluating only on archived imagery [63].

#### 3.5.1. What Running in the Field Actually Costs

Three costs recur in the deployed studies, and none appear in benchmark papers. First, *squeezing a model onto camera-side hardware costs accuracy, and the loss lands unevenly*: one classifier fell from 96% accuracy to a 0.70 F1 average after platform optimisation, with rare classes hit hardest [73], shrinking a detector cost precisely its smallest species [126], and a sub-300-milliwatt insect trigger held up on simple scenes but not complex ones [201]. Second, *the field itself degrades systems*: underwater housings fouled within a month [53], a 98-day insect tracker had to discard 87% of its raw detections as stationary false alarms [54], and the honest hardware ledgers record seven of thirty-six custom traps never working [181] and a charging fault silently consuming 154 of 814 satellite alerts [101]. Third, *in operation, error rates stop being metrics and become minutes, money and misdirected baits*: an Iridium-linked camera puts elephant alerts in Gabonese ecoguards’ hands at a median of 7.35 min for about 1200 euros plus a monthly fee [101]; a curlew network raises real-time alerts whose stated purpose is intervention speed [159]; a reserve trial couples every classification to a per-detection payment, turning false positives into a financial quantity [86]; an automated bait station decides in under two seconds whether to offer bait to an invasive canid, its threshold deliberately set low because the two kinds of error cost differently [82]; and filtering can move onto the camera itself, cutting detector runs by 84% using the sensor’s own motion signal [64].

#### 3.5.2. When Automation Pays, and Where People Stay in the Loop

Where systems run as infrastructure, the ledger is dominated by connectivity rather than by the model: a 65-camera cellular network costs more to build but 43% less over ten years than traditional trapping [122]; a feral-cat programme found the classifier alone saving 10% of costs but classifier-plus-connectivity saving 80% [140]; and an island biosecurity mesh tolerates poor precision on one species to guarantee a rodent alarm, repaying its start-up cost in twenty months [155]. At workflow scale the savings are just as concrete—five-to-eightfold processing speedups [57], twenty-three work-days of distance measurement done in under two [118], annotation of night video collapsing from over an hour per clip to under a minute [46], four-fifths of a reserve’s labelling bill automated under a deliberately cautious discard-only-on-unanimity rule [93]—but with a consistent caveat: what decides whether assistance helps is the *structure* of the errors, not headline accuracy, because a missed animal costs a human four times what a correct label saves [84], and supervised first-pass workflows cut error fourfold while halving time [120]. Camera-plus-model combinations are even beginning to replace the survey instrument itself: baited cameras out-detected live traps for all six focal rodents at a third of the effort—with the live-trap estimates the biased ones [156]—and density pipelines now run from borrowed models [195] with the distance step handled training-free to twice the previous accuracy [217].

The recurring human quantity is the hours a model’s confidence structure buys back, and the deployed studies treat the confidence threshold as an operational dial rather than a technical constant: the Arctic workflow auto-accepts labels above 0.9 confidence and retrains partly to push more images over that line [80]; the European classifier ships its threshold as a user-facing setting because partners disagree about which error matters [96]; a regional tool states feasibility as a laptop clearing a hundred videos in an afternoon [171]. Assistance does not erase expertise—experts recover abundance almost exactly where assisted novices still under-estimate [102,186]—and the tooling spectrum runs from a simple R pipeline at a third of manual cost [133] to a training-free filter whose false alarms are almost entirely livestock [132]. Privacy is handled inside the same workflows, from blurring before review [57] to removing human sequences at the honestly stated price of a third of true sequences missed [177] to transmitting labels rather than pictures [101]. The operational literature also shows the mirror image of the benchmark–practice gap—management programmes consumed as evidence that report no model accuracy at all [117,157,160]—and, where hardware and models both disappoint, the fallback is priced too: fifty hours of manual classification in one network [181], and 200,000 lake-bird images screened by hand [182].

### 3.6. Quality Appraisal of the Included Studies

Table 9 reports the criterion-level results of the quality appraisal described in Section 2.6. The six criteria (QA1–QA6) were designed for this review, because no validated quality-appraisal instrument exists for studies that develop and evaluate AI systems for ecological imagery; each is derived from an extraction item (Table 3), and together they are intended to characterise the reporting and validation profile of the corpus rather than to score or rank individual studies. The corpus is well reported but weakly validated. The reporting criteria are met almost universally: 96% of studies name their model (QA1) and 94% state where their imagery was collected (QA2), although one study in seven does not state its compute target (QA3). The criteria that concern evidence beyond the benchmark are met far less often: 62% of studies test performance beyond their training data (QA4), 58% connect their outputs to an ecological analysis or management decision (QA5), and only 35% validate their system in the field or in operation (QA6). The appraisal therefore locates the field’s weakness in validation rather than in reporting, which is the same conclusion that RQ4 and RQ5 reach from the substantive evidence.

## 4. Discussion

### 4.1. Principal Findings by Research Question

The review set out to establish to what extent AI applied to camera-trap imagery has moved from benchmark performance to ecologically usable monitoring. The answers to the five research questions are as follows.

- RQ1 (growth and venues). The field has grown 3.5-fold between 2020 and 2025, about 29% per year, and the growth continued into 2026. Output is dispersed across 340 venues with no dominant outlet, and the share published as conference proceedings rose from 30% to 41%.
- RQ2 (architectures and tasks). Detection and species classification each appear in 62% of studies, whereas behaviour recognition appears in 5%. Generic convolutional backbones and two-stage detectors dominate, MegaDetector serves as the shared detection pipeline in one study in four, and transformers (34 studies) and vision–language models (7) are now present at the margins. Almost half of the studies deliver a pipeline or tool.
- RQ3 (coverage). The literature is centred on mammals (55%), with reptiles, amphibians and aquatic taxa rarely studied, in part because passive-infrared triggers miss ectotherms. Study regions invert global biodiversity gradients: Europe (75 studies) outnumbers Asia, Australia–Oceania and Latin America combined, and Latin America appears in 10 studies.
- RQ4 (evaluation rigour). 62% of studies test performance beyond their training data, a share that has stayed between 51% and 80% every year without a rising trend. Where the penalty is measured, it ranges from about six to nearly seventy points, is driven largely by models learning the static background, and can usually be recovered with modest local adaptation.
- RQ5 (deployment and ecological use). 57% of studies never leave archived imagery and only 35% report a field trial or operational system, yet 58% connect their outputs to an ecological analysis or management decision. Only deployed or deployment-oriented studies report accuracy loss on edge hardware, degradation in the field, and the energy, labour and money a system costs; human-in-the-loop workflows are the form in which automation most often reaches practice.

Read together, these answers reveal four asymmetries:

- Task concentration. Detection and species classification each appear in about three-fifths of studies, while behaviour recognition, the task closest to ecological mechanism, appears in one in twenty (11 studies; Figure 4).
- Geographic inversion. Almost every full text names its study region (94%), and the map that results is the inverse of the biodiversity map: Europe alone outnumbers Asia, Australia–Oceania and Latin America combined, and Latin America appears in 10 studies (Figure 5).
- Uneven rigour. 62% of studies run some evaluation beyond their training data, but 30% evaluate only on a random split of the same material; the share has not improved since 2020 (Figure 6), and the penalty, when it is measured, ranges from six to nearly seventy points (Table 7).
- Minority deployment. 57% of studies never leave archived imagery and only 35% report a field trial or operational system, yet 58% connect their outputs to an ecological analysis or management decision (Table 8).

### 4.2. Comparison with Previous Reviews

The four asymmetries are not all new, and it is important to separate what this review confirms from what it adds. The predominance of species identification and counting over behavioural tasks is already visible in earlier surveys of computer vision in ecology [20]; geographic imbalance in camera-trap research is documented independently by project-level inventories [227]; and the difficulty of transferring models to new locations was demonstrated before the review period [19] and identified as a central obstacle for conservation applications [23]. On these points, the present review confirms earlier observations on a larger, systematically assembled corpus of 201 studies and turns qualitative statements into measured proportions.

The four contributions set out in Section 1.4 go beyond those observations, and the evidence qualifies the first three of them. The generalisation penalty documented in Table 7 is large but conditional—on the image-generation regime, the taxon, the camera geometry and the evaluation unit—and its recoveries are priced alongside it, so that a few hundred local images, scheduled retraining or a re-tuned threshold typically restore most of the lost performance (Section 3.4). That the share of studies testing generalisation has not risen since 2020 shows that awareness of the transfer problem has not yet changed evaluation practice. The deployment ledger (Table 8) shows that connectivity and maintenance, rather than the model, dominate the cost of a deployed system, and that edge optimisation costs accuracy unevenly, at the expense of rare classes.

### 4.3. Implications for Practice and Research

For practitioners, the evidence supports four recommendations: start from a model trained on imagery from many locations rather than from a model trained at a single site; budget for a few hundred locally annotated images to adapt it; judge it on the ecological estimate the project needs, not on a headline accuracy; and keep a human in the loop for rare and look-alike species, where errors concentrate. Where systems are to run as infrastructure, connectivity and maintenance, rather than the model, should dominate the budget.

For researchers, five practices would most change what the next review can conclude: state the architecture and the study location in the abstract as well as in the Methods, so that they are recoverable from indexed fields; make one honest generalisation test standard—a held-out camera reported beside the within-training figure—which is now cheap to pass, because a few hundred local images typically repair a failure; evaluate at the ecological unit, per event or estimate rather than per image; adopt the deployed minority’s habit of reporting degradation, hardware, hours and money as first-class results; and aim new effort at the blind spots—behaviour recognition, ectothermic taxa and the biodiverse regions that the coverage map inverts—where a competent system is worth most.

## 5. Limitations

This review searched a single database (Scopus; Web of Science and other databases were not searched), in English only. This was a scope decision as much as a limitation, since the search already yields 637 records, full-text reading of all 231 retrievable reports and a 201-study synthesis, at the upper end of what a review that reads every retrievable full text can carry. Its cost is that Scopus’s coverage of computer vision proceedings is incomplete, and the rising conference share (Section 3.1) means that this gap grows exactly where the field is moving. The search string has two blind spots—imagery described only in modality terms, and methodological papers phrased in learning-paradigm language or bare architecture names—and any such loss is selective, removing precisely the recent methodological work that RQ2 and RQ4 characterise. The string errs safely on the other side: out-of-scope pipelines that match both blocks inflate the screening burden and the *no automated method* count in Figure 1 but do not bias what is included.

Screening was single-reviewer with AI pre-classification; the seeded 10% audits of both sides found no disagreement, but audits sample rather than replicate, and dual independent screening would be the stronger design. Data extraction and quality appraisal were likewise performed by a single reviewer. One error class is invisible at abstract level: camera-trap vocabulary over imagery of other provenance, of which the 637 records contain a web-collected example [228]. Twenty-four reports could not be obtained and are absent from the synthesis.

The RQ2–RQ5 distributions rest on a documented keyword parse of free-text extraction fields into categories, with the small categories hand-checked. The parse is reproducible but not infallible: task and architecture categories overlap by design, region counts credit a study to every region it names, and the single-category fields (evaluation design, deployment status, compute target, data source and ecological use) required hand rulings for about one record in nine. The quality appraisal uses criteria derived from the extraction items rather than a validated risk-of-bias instrument because no such instrument exists for studies that develop and evaluate AI systems for ecological imagery without estimating an effect.

## 6. Conclusions

This systematic review of 201 studies published between 2020 and 2026 asked to what extent AI applied to camera-trap imagery has moved from benchmark performance to ecologically usable monitoring. Its answer is that the field has largely solved recognition within the data it is trained on but has only partly reached ecologically usable monitoring. Seven conclusions follow.

1. The field is growing quickly and changing venue. Output grew by about 29% per year, and the rising share of conference proceedings means that future reviews must search computer science sources as well as ecological ones.
2. Within-dataset performance is no longer the bottleneck. Modern convolutional, detector, transformer and edge-optimised models report roughly 80–99% on their own test data (Table 6).
3. Generalisation is the bottleneck, and its testing has not improved. Performance falls by six to nearly seventy points beyond the training data, yet only 62% of studies test for it, a share unchanged since 2020. A held-out-camera test should become a reporting standard.
4. The penalty is largely recoverable at low cost. Broad pretraining followed by adaptation with a few hundred local images, or scheduled retraining, restores most of the lost performance.
5. Ecological adequacy is decided at the estimator. Occupancy and density estimates tolerate moderate label error, but high false-positive rates can bias them severely; semi-automated workflows retain most of the labour saving while protecting the inference.
6. Deployment remains a minority, and its costs are real. Only 35% of studies reach the field; for these, connectivity and maintenance dominate cost, and edge optimisation degrades rare classes first.
7. Coverage gaps define the research agenda. Behaviour recognition, ectothermic taxa and the biodiverse tropics, Latin America in particular, are where new systems would add most ecological value.

## Figures and Tables

**Table 1:** Search strategy: concept blocks, keywords and limits applied in Scopus (TITLE-ABS-KEY fields).

Component	Keywords/Setting	Role
Concept 1: camera trapping (instrument)	“camera trap*” OR “camera-trap*” OR “trail camera*” OR “camera trapping”	Required concept; spelling variants and the trail-camera synonym
Concept 2a: field-level terms	“deep learning” OR “machine learning” OR “computer vision” OR “artificial intelligence”	Studies that name the discipline of the method
Concept 2b: method-family terms	“neural network*” OR “convolutional” OR “YOLO” OR “transformer”	Studies that name only the model family
Concept 2c: generic terms	“automated” OR “automatic”	Automated pipelines not described in machine learning vocabulary
Combination	Concept 1 AND (2a OR 2b OR 2c)	Every record must concern camera traps and automated analysis
Limits	PUBYEAR 2020–2026; language English	No taxon, subject-area or document-type limits
Database and date	Scopus, 6 August 2026	637 records retrieved

**Table 2:** Inclusion (IC) and exclusion (EC) criteria. The definitions in the text govern borderline cases.

Dimension	Inclusion Criteria	Exclusion Criteria
Imagery	**IC1.** Motion-, heat- or time-triggered cameras, or continuously recording cameras with automated image-content triggering, deployed unattended to observe free-ranging animals	**EC1.** Captive surveillance (laboratories, zoos, farms); web-, museum- or repository-sourced collections; provenance indeterminate after full-text reading (not camera-trap or provenance indeterminate)
Automated inference	**IC2.** Algorithmic detection, classification, localisation, counting or measurement of image content	**EC2.** Image management, independence filtering, spectrogram generation, triggering hardware; learning steps that consume coordinates or manual labels rather than images, e.g., Maxent (no automated method)
Tasks	**IC3.** Detection; species classification; individual re-identification; segmentation; tracking; counting; behaviour recognition	**EC3.** Analyses of the audio channel (acoustic monitoring) unless coupled to automated image analysis; generic computer vision with no animal application
Contribution types	**IC4.** Model development and evaluation; imagery datasets and benchmarks; augmentation and annotation methods; calibration and preprocessing; software tools and pipelines; deployment and edge computing	**EC4.** Bibliographic inventories, occurrence databases and geodata; editorials, notes and letters; reviews (review or editorial)
Subjects	**IC5.** Wild, free-ranging animals, including wild agricultural pests	**EC5.** Livestock and companion animals
Publication	**IC6.** Peer-reviewed journal article; published 2020–2026; English	**EC6.** Conference papers, book chapters, data papers and short surveys (recorded for RQ1, not synthesised; not a journal article). **EC7.** Full text unobtainable after retrieval attempts

Note: Criteria categories are shown in **bold**.

**Table 3:** The fourteen data items extracted from every included study.

#	Item	Content	Serves
1	Eligibility	Confirmed or reversed at full text	Selection
2	Reversal reason	Reason, where eligibility is reversed	Selection
3	Task(s)	Monitoring task(s) addressed	RQ2
4	Architecture family	CNN, YOLO, R-CNN, transformer, etc.	RQ2
5	Architecture named	Specific model(s), verbatim	RQ2
6	Taxa focus	Taxonomic group(s) studied	RQ3
7	Study location	Region of image collection, as stated	RQ3
8	Data source	Own deployment, public benchmark, or both	RQ3
9	Generalisation test	Strongest evaluation design beyond training data	RQ4
10	Headline metric	Principal performance figure, as the authors report it	RQ4
11	Deployment status	Archived imagery, released tool, prototype/field trial, operational	RQ5
12	Compute target	Workstation/server, cloud, edge/embedded, not stated	RQ5
13	Ecological use	None, feeds ecological analysis, informs management	RQ5
14	Note	Anything anomalous, free text	—

**Table 4:** Records identified per year, by Scopus publication type.

Publication Type	2020	2021	2022	2023	2024	2025	2026 ^1^	Total
Journal articles	29	33	49	44	71	83	70	379
Conference proceedings	14	12	11	31	43	68	21	200
Other document types	4	5	6	8	6	15	14	58
Total	47	50	66	83	120	166	105	637

^1^ 1 January to 6 August 2026 only. Journal articles are Scopus document type *Article*; conference proceedings are *Conference paper* and *Conference review*; other document types comprise book chapters, reviews, data papers, editorials, books, notes, letters, short surveys and one retraction notice.

**Table 5:** Architecture families documented at full text in the 201 included studies (RQ2).

Architecture Family	*n*	Representative Members
Generic CNN backbones	130	ResNet, VGG, EfficientNet, Inception, MobileNet, DenseNet, ConvNeXt, Xception
R-CNN family	82	Faster/Mask R-CNN, including MegaDetector
YOLO family	64	YOLOv2–v10 and derivatives
MegaDetector (named)	52	De facto shared detection pipeline
Transformers	34	ViT-, Swin- and DETR-based detectors and classifiers
Classical ML/CV	32	Background subtraction, frame differencing, SIFT matching, SVM, random forest
Vision–language/foundation	7	CLIP-style, SigLIP and adapted multimodal models
No architecture named	9	4% of included studies

192 of 201 studies (96%) name at least one architecture. A study may use more than one family (for example, MegaDetector for detection and a CNN classifier for species), so rows overlap and do not sum to 192. Families were assigned by a documented keyword parse of the extraction fields *Architecture family* and *Architecture named*.

**Table 6:** Reported performance of representative modern methods in the included studies (RQ2). Values are on each study’s own metric and dataset and are not directly comparable across rows.

Family	System and Task	Reported Performance
Generic CNN	Six architectures, species classification, 36 locations [34]	95.6% top-1 at trained locations; 68.7% at unseen locations
Generic CNN	ResNet50 with temperature–date–location fusion [142]	Accuracy 98.4% (image only) to 98.9% (with metadata)
Generic CNN	ConvNeXt, 88-class European mammal and bird classifier [135]	97.91% mAP (validation); 90.39% and 82.77% mAP at two held-out sites
Generic CNN (edge)	Custom 66 KB INT8 CNN, smart bait dispenser [198]	Recall 96.2%, F1 95.5%; <0.5 s inference on all four devices
Generic CNN (edge)	MobileNet v1 on Raspberry Pi, bait station [82]	Field accuracy 90%, specificity 96%; image processed in <2 s
R-CNN	MegaDetector (Faster R-CNN) zero-shot, three Bavarian regions [127]	Accuracy 96.0% (animal), 93.8% (person); daily detections vs. manual r=0.996
R-CNN	Faster R-CNN, Chernobyl Exclusion Zone [92]	81.31% image-level accuracy; 50.88% event-level success at held-out cameras
YOLO	YOLOv6-N variant on Jetson Xavier NX [126]	mAP_0.5_ 88.3% at 8.9 MB; 85.3% after INT8 quantisation at 6.15 ms/image
YOLO	YOLOv10x, real-time curlew alert network [159]	Live F1 95.05% and 96.03% (curlew and chick classes)
YOLO	YOLOv5 detector–classifier moved to a new habitat [181]	Accuracy 0.96 at the source location; 0.47 (MCC −0.01) at the new location
Transformer	Hybrid detector–ViT classifier, 45 species [210]	85.27% in distribution; 58.24% zero-shot cross-dataset, the smallest drop of six architectures
Vision–language	CLIP on Snapshot Serengeti attributes [115]	0.29 mAP zero-shot; 0.60 after fine-tuning; vision-only baseline 0.68
Vision–language	Multimodal LLM, zero-shot behaviour classification [164]	86.39% macro F1; SigLIP falls from 85.67% to 10.57% with bare-label prompts
Vision–language	Cross-continental transfer [215]	84.77% on African imagery; 16.17% on American imagery
Metric learning	Real-time individual re-identification [226]	Top-1 80–100% across six datasets; >10 images/s on a $200 edge device

**Table 7:** The generalisation penalty as reported by included studies that measure it (RQ4), with within- and beyond-training performance on each study’s own metric.

Study Design	Within-Training Performance	Beyond-Training Performance
Six architectures, 47,279 images, 36 locations [34]	95.6% top-1 accuracy (trained locations)	68.7% (unseen locations)
Classification across habitat disturbance within one survey [94]	Overall F1 0.77 with detector-cropped images (0.47 whole images)	Mean F1 0.32 (new cameras, same disturbance); 0.27 (unseen disturbance); cropping recovers unseen-level F1 to 0.73
Faster R-CNN in the Chernobyl Exclusion Zone, twelve held-out cameras from an earlier deployment period [92]	81.31% image-level accuracy (train–test cameras, confidence 0.9)	50.88% event-level success under the strictest criterion (correct species and count)
Vision–language model, cross-continental transfer [215]	84.77% (African imagery)	16.17% (American imagery); text-description fusion recovers 50.06%/39.96% (Cis/Trans)
Twenty-group classifier in a nilgai-management programme, cross-dataset test [40]	Average MCC 0.89 (south Texas deployment)	Average MCC 0.22 (CalTech dataset, southwestern USA)
Binary animal classifier at fixed Texas watering-fountain sites, 2017 model re-tested on 2019 imagery [42]	Day-model 87% sensitivity/96% specificity (2017)	81.4%/77.7% on 2019 imagery from the same sites; drift-triggered retraining restores 100%/100%
26-species Central African classifier [49]	—	Machine-label richness, activity, and occupancy (3 of 4 focal species; chimpanzee uncorrelated) matched expert labels at 227 independent stations
Fully automated global classifier (MegaDetector + SpeciesNet, no human review) vs. expert labels, three regions, 1227 cameras, ∼3.8 M images [218]	Label true-positive rates >80% for most species	Mean occupancy-rate difference 0.03–0.07 and covariate direction agreement 83–94% across 16–19 shared species per region; models resilient to false-positive rates up to ∼20%, divergence concentrated in rare and morphologically similar species
MLWIC2 base and regionally trained classifiers feeding occupancy models, three desert species, 388,108 images [124]	— (base model pretrained out of source; regional further training worth ∼9–11 points of classification success)	Image accuracy 0.821–0.956 but false-positive rates 10.2–87.9% when fully automated; occupancy bias −33 to +166%, most-supported model structures and covariate directions not recovered; semi-automated empty-culling (97.7% labour saved) matches double review
Global classifier fine-tuned on 15 Australian Wet Tropics species, held-out camera-deployment areas [212]	Macro-F1 0.964 (same-deployment test)	0.909 out-of-distribution, against 0.839 (±0.171 across seeds) for a from-scratch local model and 0.133 for the unmodified global service under its full, non-comparable label space
Bat-hibernacula pipeline (detector, segmentation, classifier ensemble), six untrained sites [90]	99.3% classification accuracy at trained sites (F1 0.97–1.00, 13 species)	96.7–98.2% where camera placement matches training (<3 m, <45°); 17.8–90.8% at atypical placements (detector F1 0.38 at the worst site); retraining the detector with 500 site-specific annotations recovers 95.5–99.9% at all six
Hybrid detector–ViT classifier trained on a 45-species Ohio dataset, zero-shot on Caltech Camera Traps [210]	85.27% (45-class in-distribution test)	58.24% zero-shot cross-dataset—the smallest drop of six architectures tested (others fall 28.8–33.4 points)
YOLOv5 detector–classifier trained at one temperate-forest location, applied to the same smart-camera design in semi-arid heathland [181]	0.96 accuracy (source-location study, same model and hardware)	0.47 accuracy, MCC −0.01 (no better than random); 0% bird precision and recall; the globally trained MegaDetector at the same sites: 0.99 precision/0.98 recall
Pretrained MegaDetector applied zero-shot to an unseen park (159,272 images, 36 cameras) [57]	— (no training at the site)	Human images 99% precision/95% recall; site-week human-event index r=0.99, mean difference 0.45% against manual classification
Pretrained MegaDetector (v4.1) applied zero-shot across three Bavarian regions (352,426 images, 159 cameras) [127]	— (no training in any region)	Accuracy 96.0% animal/93.8% person/99.3% vehicle at 95% confidence; daily detections vs. manual r=0.996; diurnal activity overlap 0.41 vs. 0.42 manual; per-site person accuracy 52–97.9% in one region
Pretrained MegaDetector applied zero-shot in Arctic tundra, split by trigger type (6224 images, 20 sites) [62]	— (no training at the site)	Motion-triggered images 94.6–95.7% correctly labelled; time-lapse images 55.7–61.6%; detection floor 60 px and maximum distance 222 m for the model, against 4 px and 2551 m for human review
Pretrained MegaDetector applied zero-shot to freshwater-lake time-lapse imagery of birds (142,801 images, two lakes) [182]	— (no training at the site; motion triggering unusable on lakes)	Recall 54.6% at 2.5% precision (0.1 confidence); recall 8.9% at 0.8; authors reject the automated workflow and screen all images manually
Pretrained MegaDetector applied zero-shot to time-lapse snake imagery, Everglades (∼1.47M images, 42 cameras) [175]	— (no snake-specific training; a few thousand internet snake crops in pretraining)	94.5% of blanks removed; snake image-level recall 17.1% at precision 0.006 (manual recall 96.2%); large (>66 cm) species matched manual review at the detection level, smaller snakes largely missed; fine-tuning on study imagery raised false positives an order of magnitude
26-class multi-partner European classifier, out-of-sample sites [96]	97.3% balanced validation accuracy	93.6% image-level/96.7% sequence-level on 213,059 images from unseen sites; but squirrel recall 0.67, ibex precision 0.45, wolf precision 0.69
88-class European mammal+bird classifier (ConvNeXt), two held-out forest test sites in Germany and Poland [135]	97.91% mAP/93.85 mean F1 (validation split)	90.39% mAP at the German site but 82.77% at the Polish site (mean F1 82.43 vs. 62.03), the larger drop attributed to poorer image quality and lower camera angles; taxonomic output heads recover 94.23%/79.92% mAP across all levels as the stated fallback for uncertain species calls
Embedded blank filter, Snapshot-Serengeti-trained, one unseen station [143]	F1 0.78 across multiple park locations	F1 0.23 (precision 0.13) at the unseen station; on-device continual learning over location-specific synthetic data restores 0.48 overall, 0.81 post-calibration
Movement-mask ensemble sequence filter, active-learning-trained on two cameras, tested at unseen sites on three continents [177]	83–86% sequence accuracy at the two training cameras (12 rounds, ∼50 annotations/class/round)	77–96% at unseen cameras (species-blind by design); a wind-exposed unseen camera collapses to 42% and one site-specific active-learning round restores 95%
Arctic small-mammal box-camera classifier (ResNet50, trained from scratch), transferred to two new study areas and a modified trap type [80]	98.2% validation accuracy (original network)	82.1% on the out-of-sample areas and 90.7% on the new trap type; retraining on 3276 of >400,000 new images restores 94.8% and 97.5%, and lifts the share of labels above 0.9 confidence from 73.7 to 94.9%
Temperate-trained video classifier applied zero-shot to tropical island ungulates (Guam) [153]	— (no training on the region; roe deer/boar training species morphologically similar)	Accuracy 78.2–84.2%, F1 ≥ 0.90 for deer and pig; the tropical-background African model manages only 39.3–67.4% on the same videos, so target similarity outweighs background similarity
Insect-trap detector fine-tuned on a published DIY-trap dataset, re-evaluated on the authors’ own 27-plot grassland deployment [208]	96.1% mAP@0.5, 96.3% precision, 93% recall (source test set)	25.8% mAP@0.5, 41.6% precision, 29.1% recall on leakage-controlled field imagery; attributed to community shift, vegetation ingrowth and the removal of near-duplicate frames between splits
Ultra-lightweight MCU insect triggers (94k–411k parameters), field datasets unseen in training [201]	AUROC 0.918–0.964 on the withheld test set	AUROC 0.818–0.872 on one unseen field dataset but 0.588–0.691 on the other, cluttered-background one—out-of-domain success limited to simple scenes
Bear pose estimation + MLP bipedalism classifier, test set exclusively from seven new 2023 camera stations [197]	— (no station shared with training)	96.1% bipedalism-vs-quadrupedy accuracy on predicted keypoints (97.4% on ground-truth keypoints); residual errors confined to posture-transition and sitting edge cases
GBIF-trained hierarchical insect classifier (1.12 million web-scraped images, 1127 taxa; missing-label-aware loss), applied without further training to the authors’ own light-trap camera deployment in Costa Rica and Ecuador [203]	Balanced accuracy 98.6%/94.6%/85.7%/84.3% order through species (GBIF validation split)	Field balanced accuracy 98.3%/96.2%/93.3%/79.2% with recall 97.0%/92.7%/86.8%/58.5% order through species; species precision 29.2% over all predictions vs. 84.4% on confirmed taxa; genus precision approaches 100% only above 500 × 500 px crops, and ∼21% of field crops fall below the consistently identifiable size—the web-to-field drop concentrates at fine taxonomic levels and small subjects

**Table 8:** Reported deployment evidence in the included studies that provide it (RQ5), including deployment-oriented systems that were bench-measured rather than field-deployed. These papers report degradation and resource cost as first-class results; benchmark papers report neither.

System	Reported Evidence
Edge classification, three platforms [73]	96.1% accuracy before optimisation; macro F1 falling to 0.7 after, minority classes to 0.18; latency and current draw measured
Real-time re-identification [226]	40–60 cropped images/s on a laptop; >10/s on a $200 Cortex-A72 edge device, CPU-only; Top-1 80–100% across six datasets; 50% occlusion costs 9 points of Top-1 on average
Raspberry Pi Zero classification [128]	On-device classification and explainability at weighted F1 ≈ 0.95 (macro 0.87; one minority bird class at 0.16); 6 s per inference, 23.26 s per Grad-CAM visualisation; 50 mA (0.25 W) after HDMI shutdown on a solar power bank, 72-h prototype run
Cellular real-time bird classification [81]	Two years, eight feeder sites, UK and Netherlands; 42,973 images over 3/4G to server-side Faster-RCNN; 96.71% accuracy, 98.28% blank removal, sensitivity 88.79%; precision 75.32% average, spanning 35.0% (jay) to 100% (small, distant species; the stated mechanism: validation mAP 0.79 large vs. 0.46 small objects); untrained species mapped to the nearest class
Edge-optimised detector [162]	Parameter, FLOP and model-size reductions for deployment—yet evaluated only on archived imagery; the intelligent camera trap is listed as future work
INT8 wildlife detector on Jetson Xavier NX [126]	Reparameterised YOLOv6-N variant, mAP_0.5_ 88.3% at 8.9 MB; 6.15 ms/image at INT8 keeping mAP_0.5_ 85.3% under reparameterisation-aware training vs. 84.2% post-training quantisation; head pruning costs the smallest species (chevrotain AP 0.978 → 0.945); evaluated on archived imagery, the in-field system stated as the goal
24-h ectotherm video network, citizen-science-hosted [165]	27 always-on video traps over 7 months; >10^6^ videos; YOLOv5 screening saves an estimated 1300 h of labelling; <5 cameras/site for 95% detection confidence of most modelled taxa
Real-time insect tracking on a 5-W edge computer [54]	98-day unattended season at 0.33 fps; 87% of raw detections removed as stationary false positives; 89% track precision, one class at 0.39
27-trap solar insect network, three grassland regions [208]	2654 trap-days over one season; on-device YOLOv5n gated by optical-flow motion detection (3.15 → 2.70 W); battery thresholds throttle recording windows; failures, overcast and vandalism gaps absorbed by two-week aggregation, each interval retaining ≥21 of 30 mowing events
Underwater camera-trap network, $125/unit [53]	5-month unattended marine deployments; biofouling degrades image quality and model accuracy within 1–2 months; analysis restricted to first-month predictions
MegaDetector-assisted recreation-ecology workflow [57]	506% overall/840% manual-component speedup on 159,272 images; 40% of volume fully automatic; privacy blurring before human review
Raspberry Pi video camera trap, in-capture-loop filtering [64]	PIR trigger replaced by hardware H.264 motion vectors (<0.4 ms/frame vs. 1.2 s YOLOv4-tiny inference); 84.1% fewer model runs; 2.20–6.25 W; multi-day field deployments
Edge classification, three platforms [73]	96.1% accuracy before optimisation; macro F1 falling to 0.7 after, minority classes to 0.18; latency and current draw measured
Real-time 3/4G reserve-wide trial with per-detection payments [86]	27 cameras, 400 km^2^, 10 months; 96.38% sensitivity/87.14% precision over 12 taxa; GBP 185.20 auto-transferred over 18,520 detections; lion and cheetah precision ≈54/59% as the scheme’s weak point
Edge bait station actuating pest control [82]	MobileNet v1 on Raspberry Pi; image processed <2 s, bait offered in 9.81 s; field accuracy 90%, specificity 96% at a deliberately low 5% threshold; 19 baits offered, 7 taken by target canids
Autonomous 4G camera network, 65 cameras, Natura 2000 site [122]	Solar-powered, no battery/SD visits (pilots autonomous 746 days); end-to-end pipeline to AI species ID; establishment +131% but annual costs −51%, 10-year total −43% (>500,000 euros) vs. traditional trapping; focal-species recall 0.42–0.50 keeps a human in the loop
Solar-autonomous insect camera trap, ∼700 euros [139]	Five traps, 1919 h over a full season at ∼4.4 W; on-device detection and tracking stores only cropped detections (5–10 KB vs. 0.5–1 MB per frame); curated-test top-1 0.972 falls to 0.2–0.49 for heterogeneous classes on 97,671 real-world images
Self-calibrating blank-filter camera trap, ∼$100 [143]	On-device transfer + continual learning in 8–15 min at <0.6% of battery; F1 0.48 at an unseen station vs. 0.23 without calibration and 0.43 for zero-shot MegaDetector
Operational island-biosecurity mesh network, AI triage [155]	10 radio-linked cameras, 17 months; rodent alarm channel at 0.998 precision/0.934 recall reviewed within 24 h; downtime 4% of camera-days vs. 10% for the SD-card fleet it replaced, whose cards waited 56.5 days on average for review; annual maintenance $896 vs. $7842, break-even at 20 months
Edge-evaluated insect pipeline, two full seasons [154]	12 UV traps, >5.7 million images, >3000 nights; Jetson Nano processes at 26.3 J/image (RPi4 148.9 J), near-real-time at ∼0.5 fps; 2-min time-lapse reproduces tracked counts, enabling on-trap processing; three edge units uploaded abundance statistics for three months
Edge-evaluated metric-learning false-trigger filter [190]	0.076 s/image on a Jetson Nano at 80.17% accuracy/19.48% FPR—36.7% faster than the best YOLO comparator at comparable recall, trained from class labels only (no bounding boxes); archived imagery, communication-equipped traps the stated target
MCU insect trigger on a ∼£20 ESP32-S3 board [201]	Int8-quantised models with no significant accuracy loss; <300 mW at maximum throughput, ∼185 mW at half; lightest models exceed 8 frames/s, the most accurate 2; zero trigger-to-capture latency by retrospective design—but bench-measured only, and idle draw cannot match a PIR sensor’s ≪1 mW
Smart bait-dispenser edge benchmark, four devices × six quantised CNNs [198]	66 KB INT8 custom CNN at recall 96.2%/F1 95.5%, beating four fine-tuned backbones; all devices <0.5 s mean inference; Portenta H7 selected at 0.185–0.19 W idle/inference, with an ATtiny85 motion-wake switch (6–30 *μ*W sleep) proposed for multiday vaccination campaigns—bench-measured, field trials future work
Custom smart video traps for small fauna, 36 units, semi-arid heathland [181]	Downward-facing continuous video with on-board foreground-detection capture, solar-powered; 7 of 36 units never worked and 22 stopped after 66 video segments—failures first-class; only these traps detected nocturnal ectotherms and invertebrates (frogs, small reptiles, spiders, scorpions: zero on the paired commercial cameras)
Real-time 3/4G curlew alert network, 11 nesting sites [159]	Six-week live deployment (May–June 2024), 1072 images; solar cellular traps → SMTP → GPU-server YOLOv10x inference; alerts above 0.30 confidence for curlew and chick classes; live F1 95.05%/96.03% with 100% curlew precision; sub-threshold images discarded as blanks (98.28% claimed); residual errors concentrated in curlew–pheasant confusion, camera placement flagged as critical for chicks

**Table 9:** Quality-appraisal criteria and results for the 201 included studies. Each criterion is answered Yes or No from the extraction item shown.

Code	Criterion (Extraction Item, Table 3)	Yes, *n*	Yes, %
QA1	Is at least one model architecture named? (item 5)	192	96
QA2	Is the region of image collection stated? (item 7)	189	94
QA3	Is the compute target stated? (item 12)	172	86
QA4	Is performance evaluated beyond the training data (cross-site, cross-dataset, temporal or cross-camera)? (item 9)	124	62
QA5	Are outputs linked to an ecological analysis or management decision? (item 13)	117	58
QA6	Is the system validated in a field trial or operational deployment? (item 11)	70	35

## Data Availability

The original contributions presented in this study are included in the article. Further inquiries can be directed to the corresponding author.
